# Development and Application of an eDNA Method to Detect the Critically Endangered Trinidad Golden Tree Frog (*Phytotriades auratus*) in Bromeliad Phytotelmata

**DOI:** 10.1371/journal.pone.0170619

**Published:** 2017-02-15

**Authors:** Sarah Brozio, Chloe Manson, Eleanor Gourevitch, Thomas J. Burns, Mark S. Greener, J. Roger Downie, Paul A. Hoskisson

**Affiliations:** 1 Strathclyde Institute of Pharmacy and Biomedical Sciences, University of Strathclyde, Glasgow, United Kingdom; 2 School of Life Sciences, Graham Kerr Building, University of Glasgow, Glasgow, United Kingdom; University of Hyogo, JAPAN

## Abstract

The use of environmental DNA (eDNA) to monitor rare and elusive species has great potential for conservation biology. Traditional surveying methods can be time-consuming, labour-intensive, subject to error or can be invasive and potentially damaging to habitat. The Trinidad golden treefrog (*Phytotriades auratus*) is one such species that would benefit from such an approach. This species inhabits the giant bromeliad (*Glomeropitcairnia erectiflora*) on two peaks on the Caribbean island of Trinidad. Traditional survey methods for this species have required the destruction of the giant bromeliad, which is the only known habitat of this frog. Here we described the development of an eDNA PCR-based assay that uses water drawn from the water-filled phytotelmata of the giant bromeliad along with the use of a synthetic DNA positive control that can be easily amplified in the bacterium *Escherichia coli*. The assay can detect to a DNA concentration of 1.4ng. Sampling of 142 bromeliads using this method revealed 9% were positive for *P*. *auratus* DNA. These data suggest that eDNA methods also have great potential for revealing the presence of elusive species in arboreal habitats.

## Introduction

The extraction and identification of DNA from environmental samples has recently shown great potential for the monitoring of endangered and elusive species[1,2]. Traditional surveying methods in zoology for the determination of a particular species are generally time consuming, highly labour-intensive and vulnerable to observer error[1]. Complementary approaches to identify elusive or rare species have been sought for a long period to aid in surveying. Environmental DNA (eDNA) is nuclear or mitochondrial DNA that is released from an organism in to the environment. The analysis of eDNA has shown great potential in the field of conservation biology over a range of habitats [1–4]. Sources of eDNA include faeces, mucous, gametes, shed skin and carcasses and as such can report on the presence of a given species in a habitat, thus finding application in a range of ecological studies [1]. In aquatic environments eDNA is dispersed within the water and can persist for between one and 21 days depending on the prevailing environmental conditions and has successfully been used in invasive species monitoring[5], disease monitoring [6] and amphibian conservation monitoring [3].

The Trindad Golden tree frog *(Phytotriades auratus*) is considered critically endangered based on its restricted geographical range [7]. Historically reported from the two highest peaks in the Northern Range of the island of Trinidad (El Tucuche and Cerro del Aripo) at elevations greater than 600 m above sea level. Two other peaks in Trinidad (Morne Bleu and Chaguaramal) may have previously harbored populations of the species although suitable vegetation is no longer present at these sites [7]. Clarke et al [8] searched for *P*.*auratus* in three species of large bromeliad at the summits of El Tucuche and Cerro del Aripo in 1993 and 1994 and found them only in *Glomeropitcairnia erectiflora*, with the main factor determining their suitability appearing to be the large volumes of water these bromeliads could hold. This suggests that this species may be acutely susceptible to habitat decline and potentially to future impacts of climate change. Recently a population of *P*. *auratus* was identified on Cerro Humo, Península de Paria, Sucre State, Venezuela—perhaps unsurprising given the connection in geological terms of Trinidad to the Guiana Shield [9]. However, no molecular phylogenetic analysis has been published thus far to unequivocally assign taxonomic status of this population as being genetically identically to those on Trinidad.

The introduction of the Environmental Management Act (EMA) in 2013 afforded protection to the Northern Game Sanctuary, encompassing El Tucuche in Trinidad and provides protection to the habitat of *P*. *auratus* in this area [9]. Current surveying methods for *P*. *auratus* require the sacrificial survey of *G*. *erectiflora* to ascertain the presence or absence of the adults or young of *P*. *auratus*. This approach is clearly undesirable given the slow growth of *G*. *erectiflora* and limited range and habitat of both species in Trinidad and beyond. Development of an eDNA based method for surveying *P*. *auratus* offers great potential for success given that all reported sightings of the species are from this plant species only, with phytotelmata holding up to 700 ml of water and tadpoles of this species are only found in these bracts [7].

Here we developed a rapid, robust, non-invasive, non-destructive survey method for *P*. *auratus* using eDNA collected from *G*. *erectiflora* phytotelmata as a template, followed by standard PCR, allowing rapid assessment of the presence of *P*. *auratus* DNA in environmental samples. This method has great potential for more extensive assessment of the *P*. *auratus* population on Trinidad and can be adapted for species with similar habits.

## Materials & Methods

### Ethics statement

"Wildlife Section, Forestry Division, of the Government of Trinidad and Tobago for issuing Special Game Licenses under the Conservation of Wildlife Act"

### Study site and sample collection

Water samples from the phytotelmata of epiphytic *Glomeropitcairnia erectiflora* bromeliads were collected close to the summit of El Tucuche, Trinidad. Water samples were collected in at least 50 ml volumes into sterile suction operated, mucus specimen trap sets (Pennine Healthcare). Each sample was collected using a freshly opened sterile Mucus trap and fresh gloves to minimize cross-sample contamination. Samples were immediately placed in a cool box containing ice for transport to the laboratory at the University of the West Indies. Samples were filtered (see below) before storing at -20°C. Samples were later transported to the UK on ice and transferred to -80°C.

### Extraction of eDNA from water samples

Water samples (50 ml) were filtered with 0.2μM Whatman cellulose nitrate membrane filters to collect biomass from the samples (in the laboratory at the University of the West Indies) and stored in 95% ethanol. Membranes stored at -80°C were transferred to 15 ml disposable tubes and material removed from the filters by vortexing in 200 μl of 10 mM Tris HCl (pH 8)/0.1% EDTA (w/v)/ 0.5% SDS (w/v) buffer (Sigma-Aldrich) for 1 min. To each tube, 0.1ml of Proteinase K (300μg/ml in the same buffer) was added and the samples were incubated at 50°C for 1h. At this point the membrane was removed and DNA was extracted through the addition of 0.3ml buffered phenol (10mM Tris HCl [pH8], 1mM EDTA; Sigma-Aldrich), followed by vortexing for 1 min [10]. Samples were subsequently centrifuged for 5 minutes at 12470 x *g* to separate the phases. The upper phase (aqueous phase) of each sample was removed and DNA was precipitated with twice the volume of 95% ethanol, by incubating on ice for 15 min. DNA was pelleted by centrifugation for 15 min at 12470 x *g* at 4°C. The supernatant was removed and the DNA pellet was washed with 70% ethanol followed by air drying. Pellets were resuspended in 50μl of distilled H_2_O.

### Synthesis of *P*. *auratus* CytB positive control DNA

To establish the eDNA assay for *P*. *auratus* a positive control DNA sample was required. To negate the need to sacrifice frogs or tadpoles of the endangered *P*. *auratus*, the 390 bp cytochrome *b* partial gene fragment for T521 (GenBank accession number DQ403734; Jowers *et al*.,[7]) was synthesized by Eurofins MWG (Ebersberg, Germany) in the vector pEX-A to yield the vector pGTF-CytB (S1 Data File https://dx.doi.org/10.6084/m9.figshare.4547344.v1). This enabled the replication of pGTF-CytB containing the *cyt*B gene fragment from *P*. *auratus* to be amplified in the bacterium *Escherichia coli*. This facilitated the production of large amounts of high quality positive control DNA for *P*. *auratus*. The *E*. *coli* strain DH5α (Novagen) was used for routine propagation of pGTF-CytB in L-Broth containing 50 μg ml^-1^ of carbenicillin (Melford Labs) according to standard protocols [11]. Plasmid DNA was extracted using Genomic DNA extraction kit (Qiagen).

### Polymerase Chain Reaction (PCR) procedure for eDNA

Cytochrome B DNA primers specific for *P*. *auratus* were designed based upon the cytochrome *b* sequence for voucher specimen T521, located under the GenBank accession number DQ403734 [7]. Primer sequences are as follows—GTF-F Forward Primer– 5`-CCCCTTACATCGGCACTGAC-3`and GTF-R Reverse Primer– 5`-CTCCAAGGATGTTTGGGGTGA-3`.

Control PCR analysis in order to demonstrate that PCR quality DNA had been extracted from the environmental samples and was suitable for amplification was performed using universal 16S rDNA primers (16Sar 5’-CGC CTG TTT ATC AAA AAC AT-3’ and 16Sbr 5’-CCG GTC TGA ACT CAG ATC ACG T-3’;[12].

Polymerase Chain Reaction mixes were set up as master mixes to ensure consistency for all samples, with each reaction containing 1μl Bioline Taq E polymerase, 10μl 5x MyTaq buffer (Bioline), which include dNTPs, 1μl (100 pmol/μl) each of forward and reverse primers and 36μl nuclease-free H_2_O. Each reaction contained 1μl of template, with DNA concentrations that ranged from 0.5–15 μg/μl depending on the sample. All PCR reactions were conducted with appropriate positive and negative controls. All samples were examined by agarose gel electrophoresis (1.5% agarose in 1 x TBE buffer) according to standard methods, staining with ethidium bromide[10].

## Results

### Collection of samples from Glomeropitcairnia erectiflora

**Fig 1:**
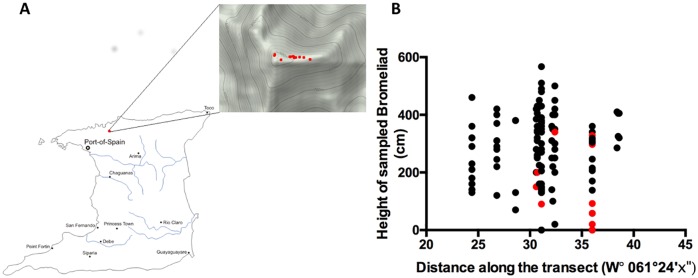
Position of *P*. *auratus* sampling locations. **(A)** Map of Trinidad showing the location of El Tucuche on the island of Trinidad, West Indies. The inset shows the location of the sample GPS coordinates along the summit (red points). The map was constructed from http://www.d-maps.com/carte.php?num_car=28037&lang=en and GPS coordinates plotted using HamsterMap. **(B)** Scatter plot of the samples as GPS coordinates plotted against height above ground of bromeliad phytotelmata sampled. Red points indicate those samples that were PCR positive for *P*. *auratus* DNA. (Supplementary Table 1: GTF Field and PCR raw data: https://dx.doi.org/10.6084/m9.figshare.4547338.v1).

Sampling of water from phytotelmata of the epiphyte *G*. *erectiflora* was conducted around the summit of El Tucuche in the Northern Range of Trinidad between June and August 2015 (Fig 1A and Supplementary Table 1: GTF Field and PCR raw data: https://dx.doi.org/10.6084/m9.figshare.4547338.v1). Samples were collected non-invasively through the use of mucus specimen trap sets (Pennine Healthcare), which allowed a tube to be inserted deep in to each phytotelma and a 50 ml water sample to be removed. A total of 142 bromeliads were sampled, with one to three bracts sampled per plant. Bromeliads were sampled at an altitude of between 870 and 910 m above sea level. Samples from *G*. *erectiflora* phytotelmata were located between zero and 5 m above the ground. Samples collected at ground level were from *G*. *erectiflora* located on fallen branches. No frogs or tadpoles were encountered in any phytotelmata that were visually examined; however, this species is known to hide deep between the leaves when disturbed (Greener, Personal Communications;[13]).

### Development of an eDNA assay for *Phytotriades auratus*

**Fig 2:**
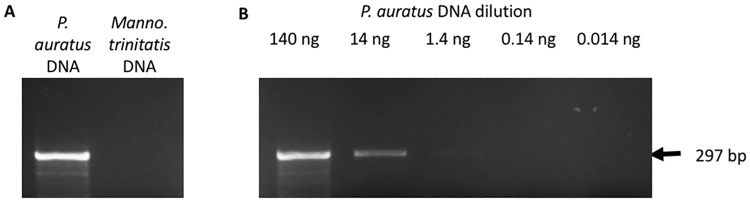
Specificity and detection limits of the *P*. *auratus* PCR assay. **(A)** Using *P*. *auratus* DNA as a template yields a single amplicon of 297 bp. Using *Mannophryne trinitatis* genomic DNA as a template yielded no amplicon. **(B)** Dilution series of pGTF-CytB DNA from 140 ng per reaction to 0.014 ng per reaction. Amplicons were apparent by agarose gel electrophoresis and ethidium bromide staining down to 1.4 ng of DNA per reaction.

A PCR based assay was developed based on primers designed against the *P*. *auratus* Cyt*B* sequences deposited in GenBank (DQ403734, DQ403735, DQ403736, DQ403737, DQ403738, DQ403739 and DQ403740 [7]). The amplification conditions were optimised using a synthetic DNA standard cloned into a plasmid that can replicate in the bacterium *Escherichia coli*. The optimal annealing temperature for the primers (see materials and methods) was found to be 50°C and 30 cycles of amplification were suitable to generate the predicted 297 bp amplicon (Fig 2A). Sequencing of the PCR product confirmed the expected sequence. Annealing temperatures of 48 and 52°C yielded no amplicons (Supplementary Figure 1: https://dx.doi.org/10.6084/m9.figshare.4547341.v1). The primers were specific for *P*. *auratus CytB* sequences, as PCR assays using the Trinidad stream frog, (also occurring on the slopes of El Tucuche) *Mannophrynne trinitatis* DNA as a template was found not to yield any amplicons (Fig 2A). A dilution series of pGTF-CytB was produced that allowed PCRs to be performed to investigate the detection limit of the assay. Fig 2A shows that as little as 1.4 ng of *P*. *auratus* DNA could be detected using 30 amplification cycles within the PCR using serial dilutions of the positive control DNA (Fig 2A).

### Determination of *Phytotriades auratus* from water collected in the phytotelmata of *Glomeropitcairnia erectiflora* from El Tucuche, Trinidad

**Fig 3:**
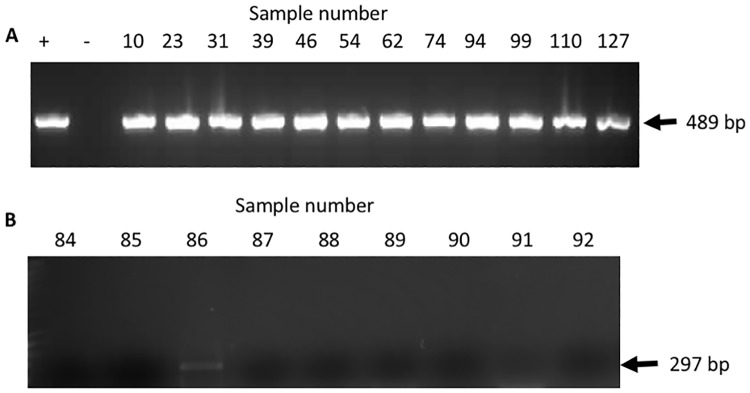
Determination of *P*. *auratus* DNA in the phytotelmata of *Glomeropitcairnia erectiflora*. **(A)** Electrophoretic analysis using DNA extracted from phytotelmata water samples as a template and Universal 16S rDNA primers (See Materials and Methods). + is a positive control consisting of *Mannophryne trinitatis* genomic DNA.–is a ‘No DNA’ negative control. **(B)** A representative electrophoretic analysis of a positive sample (Sample 86) from the phytotelmata water showing the presence of *P*. *auratus* DNA.

Total DNA was extracted from water samples collected at the summit of El Tucuche. To determine if the DNA extracted was of suitable quality to allow successful PCR, a random selection of 12 total DNA samples was subjected to PCR using 16Sar and 16Sbr primers[12] (Fig 3A). All samples were found to yield the expected amplicons (Fig 3A) indicating that PCR quality DNA was extracted from the water samples.

Given that the phytotelmata water samples yielded PCR quality DNA, all water samples were subjected to PCR using the GTF-F and GTF-R primer pair to determine if there was *P*. *auratus* DNA in any of the 142 samples. Electrophoretic analysis of the PCR reactions showed 13 positive samples, a total of 9% (representative positive shown in Fig 3B). All PCR assays were performed in duplicate and identical results were obtained (Supplementary Table 1: GTF Field and PCR raw data: https://dx.doi.org/10.6084/m9.figshare.4547338.v1). Samples positive for *P*. *auratus* were found to be from bromeliads found between zero (fallen plants) and 3.6 m above ground at elevations between 870 and 908 m above sea level (Fig 1B and Supplementary Table 1: GTF Field and PCR raw data: https://dx.doi.org/10.6084/m9.figshare.4547338.v1).

## Discussion

The golden tree frog (*Phytotriades auratus*) is a highly elusive species, with a constrained range in Trinidad (by altitude and breeding site availability) which is likely to be significantly affected by climate change [7]. Current survey methods require destructive sampling of the only known breeding sites of *P*. *auratus–*the phytotelmata of the giant bromeliad (*Glomeropitcairnia erectiflora*)[7,13]. Visual sampling for elusive species is time consuming, labour-intensive and subject to observer error [1] and in the case of *P*. *auratus* destructive, as *G*. *erectiflora* needs to be removed from branches and the leaves separated, thus destroying *P*. *auratus* habitat. To avoid damage to habitat we have developed a non-destructive, non-invasive method to utilise eDNA from the water filled phytotelmata of *G*. *erectiflora* to survey for this species. Recent work on using eDNA to survey for amphibians has shown the value of such methods as sensitive and efficient tools to detect elusive or difficult to survey species and is becoming the preferred method of surveying given the cost and time effective nature of its application [3,14–16]. The decision to use standard PCR to detect eDNA in this study was based on the need to prove presence or absence of the frogs. It has been suggested that qPCR can give quantitative measurements of population size [2], but these approaches have several potential pitfalls in terms of their application and execution related to the mass of the organism, the quality of the water and the release rate of DNA containing material by the target species[1]. There are advantages to using qPCR approaches for detecting eDNA such as increased sensitivity [2], however the relatively small volumes of water present in phytotelmata and the ability to use this method in laboratories without sophisticated qPCR machines makes standard PCR more useful in this situation.

This study found, using PCR, that around 9% of samples were positive for *P*. *auratus*. Whilst it is impossible to identify the life stage of *P*. *auratus* that resulted in the positive eDNA results, it does indicate that this method has great potential for an expanded study to collect increased numbers of samples and to also investigate other sites where *P*. *auratus* has been recorded previously or may represent suitable habitat—Cerro del Aripo, Morne Bleu and Chaguaramal [7,9]. Clarke et al [8]found *P*.*auratus* in nine of 25 *G*. *erectiflora* sampled in 1993 and 1994. Of these nine positives, five contained both adults and tadpoles (two adults in one case), three adults only, and one a single tadpole only. Jowers *et al* [7] found seven adult P. auratus in 20 bromeliads sampled on El Tucuche in 2003, a proportion not significantly different to Clarke et al's [8] findings, although the proportion of inhabited bromeliads on Cerro del Aripo was much lower in the more recent of these two studies. Assuming that eDNA is equally detectable whether a bromeliad is inhabited by adults or tadpoles or both, it is clear that eDNA cannot be used to determine population size, though it could be used to assess occupancy.

The method developed here for surveying *P*. *auratus* from *G*. *erectiflora* represents the first application of eDNA methodology to phytotelmata, which represent significant egg deposition and larval habitats for amphibians globally [17–19] and offers a less invasive, destructive and rapid method of surveying for an elusive amphibian species. This method and pilot study will hopefully provide a suitable framework for a more extensive and detailed studies of the distribution, population structure and habits of a critically endangered species. This work also suggests that metagenomics studies have great potential for identifying elusive species from eDNA samples. Moreover, the application of rapid DNA synthesis and amplification in standard laboratory bacteria is a novel way to generate reliable control DNA of known sequence for use in molecular ecology.

### Note added in revision

We note the publication of a paper by Torresdal *et al* [20] while we were revising our manuscript. This work also used an eDNA approach to detect *P*. *auratus* from bromeliads (23 positives from 29 eDNA samples taken) and noted that there is an additional site in Trinidad (Chaguaramal) that was DNA positive for this species.

## Supporting Information

S1 Dataset Sequence of pGTF-CytB. (PDF)
