# Supplementary material for: Development and Application of an eDNA Method to Detect the Critically Endangered Trinidad Golden Tree Frog (Phytotriades auratus) in Bromeliad Phytotelmata
Source: PLoS One. 2017 Feb 15;12(2):e0170619. doi: 10.1371/journal.pone.0170619 (PMC5310848; doi:10.1371/journal.pone.0170619)
Supplement: S1 Dataset — (PDF) [file pone.0170619.s001.pdf]

Supplementary Data File 1

Sequence of pGTF-CytB (Brozio et al., 2016)

Phytotriades auratus CytB gene fragment is the first 390 bp (in Upper case), followed by 2450 bp of pEX-A (MWG Eurofins).

Site bases (below) refer to positions from the first lower case base
Multiple Cloning Site: bases 2421-31
lac Promoter: bases 28-143
pUC origin: bases 419-1007
Ampicillin resistance ORF: bases 1178-2038
pEX-For priming site: bases 2323-2343
pEX-Rev priming site: bases 75-99

**AGCGACACAGTTATTACAAATTTACTTTCAGCTGCCCCTTACATCGGCACTGACCTGGTTCAATG
GATCTGAGGCGGATTCTCTGTCGACAATGCAACTCTCACCCGATTTTTTACATTTCACTTTATCCT
TCCATTTCTTATTGCAGGAGCATCAATGATTCATCTTCTTTTTCTTCACCAAACAGGCTCATCCAA
CCCCACAGGACTTAACTCAAACCCAGACAAAATTCCATTTCATTCCTATTATTCTTACAAAGATATT
TTCGGCTTCGCAATTTTACTTGCCTTCCTCGCAACTTTATCCACCTTCACCCCAAACATCCTTGGA
GACCCAGATAACTTTACACCAGCTAACCCATTAGTCACCCCTCCCCATATTAAACCAGAAacctgcg
gccgcaagcttggatccgaattcctgtgtgaaattgttatccgctcacaattccacacaacatacgagccggaagcataaagtgtaaagcctgg
ggtgcctaatgagtgagctaactcacattaattgcgttgcgctcactgcccgctttccagtcgggaaacctgtcgtgccagctgcattaatgaatc
ggccaacgcgcggggagaggcggtttgcgtattgggcgctcttccgcttcctcgctcactgactcgctgcgctcggtcgttcggctgcggcgag
cggtatcagctcactcaaaggcggtaatacggttatccacagaatcaggggataacgcaggaaagaacatgtgagcaaaaggccagcaa
aaggccaggaaccgtaaaaaggccgcgttgctggcgtttttccataggctccgcccccctgacgagcatcacaaaaatcgacgctcaagtca
gaggtggcgaaacccgacaggactataaagataccaggcgtttccccctggaagctccctcgtgcgctctcctgttccgaccctgccgcttacc
ggatacctgtccgcctttctcccttcgggaagcgtggcgctttctcatagctcacgctgtaggtatctcagttcggtgtaggtcgttcgctccaagctg
ggctgtgtgcacgaaccccccgttcagcccgaccgctgcgccttatccggtaactatcgtcttgagtccaacccggtaagacacgacttatcgc
cactggcagcagccactggtaacaggattagcagagcgaggtatgtaggcggtgctacagagttcttgaagtggtggcctaactacggctac
actagaaggacagtatttggtatctgcgctctgctgaagccagttaccttcggaaaaagagttggtagctcttgatccggcaaacaaaccaccg
ctggtagcggtggtttttttgtttgcaagcagcagattacgcgcagaaaaaaaggatctcaagaagatcctttgatcttttctacggggtctgacgc
tcagtggaacgaaaactcacgttaagggattttggtcatgagattatcaaaaaggatcttcacctagatccttttaaattaaaaatgaagttttaaa
tcaatctaaagtatatatgagtaaacttggtctgacagttaccaatgcttaatcagtgaggcacctatctcagcgatctgtctatttcgttcatccata
gttgcctgactccccgtcgtgtagataactacgatacgggagggcttaccatctggccccagtgctgcaatgataccgcgagacccacgctca
ccggctccagatttatcagcaataaaccagccagccggaagggccgagcgcagaagtggtcctgcaactttatccgcctccatccagtctatt
aattgttgccgggaagctagagtaagtagttcgccagttaatagtttgcgcaacgttgttgccattgctacaggcatcgtggtgtcacgctcgtcgtt
tggtatggcttcattcagctccggttcccaacgatcaaggcgagttacatgatcccccatgttgtgcaaaaaagcggttagctccttcggtcctcc
gatcgttgtcagaagtaagttggccgcagtgttatcactcatggttatggcagcactgcataattctcttactgtcatgccatccgtaagatgcttttct
gtgactggtgagtactcaaccaagtcattctgagaatagtgtatgcggcgaccgagttgctcttgcccggcgtcaatacgggataataccgcgc
cacatagcagaactttaaaagtgctcatcattggaaaacgttcttcggggcgaaaactctcaaggatcttaccgctgttgagatccagttcgatgt
aacccactcgtgcacccaactgatcttcagcatcttttactttcaccagcgtttctgggtgagcaaaaacaggaaggcaaaatgccgcaaaaa
agggaataagggcgacacggaaatgttgaatactcatactcttcctttttcaatattattgaagcatttatcagggttattgtctcatgagcggatac
atatttgaatgtatttagaaaaataaacaaataggggttccgcgcacatttccccgaaaagtgccacctgacgtctaagaaaccattattatcatg
acattaacctataaaaataggcgtatcacgaggccctttcgtctcgcgcgtttcggtgatgacggtgaaaacctctgacacatgcagctcccgg
agacggtcacagcttgtctgtaagcggatgccgggagcagacaagcccgtcagggcgcgtcagcgggtgttggcgggtgtcggggctggct
taactatgcggcatcagagcagattgtactgagagtgcaccatatgggtaccgagctcgcggccgcaagc**
